# Advanced Materials for Clinical Endodontic Applications: Current Status and Future Directions

**DOI:** 10.3390/jfb15020031

**Published:** 2024-01-26

**Authors:** Saulius Drukteinis, Sivaprakash Rajasekharan, Matthias Widbiller

**Affiliations:** 1 Institute of Dentistry, Faculty of Medicine, Vilnius University, Zalgirio 115, LT-08217 Vilnius, Lithuania; 2 Department of Paediatric Dentistry, School of Oral Health Sciences, Ghent University, B-9000 Ghent, Belgium; sivaprakash.rajasekharan@ugent.be; 3 Department of Conservative Dentistry and Periodontology, University Hospital Regensburg, Franz-Josef-Strauß-Allee 11, D-93093 Regensburg, Germany; matthias.widbiller@ukr.de

Endodontics has significantly evolved in recent years, with advancements in instruments, biomaterials and nanomaterials science playing a pivotal role [1,2]. These cutting-edge materials are transforming endodontic treatment techniques, offering enhanced properties, improved clinical outcomes and a more patient-centered approach [3]. Within the last decade, a wide variety of materials for root canal irrigation, disinfection and obturation, as well as for the management of endodontic complications, regenerative endodontic procedures (REP), endodontic surgery and pediatric endodontic treatment, have been introduced. Therefore, detailed in vitro, in vivo and clinical investigations of these materials are crucial for their scientifically based, standardized, safe and successful use in daily clinical practice [4].

The quality of root canal obturation and materials used for this purpose, including endodontic sealers, play a significant role in the success of endodontic treatments. These materials should ensure a three-dimensional seal within the root canal system, preventing re-infection and ensuring the longevity of the treatment and the survival of the endodontically treated tooth. Over the years, various endodontic sealers have been developed, each with unique properties, advantages and disadvantages [5]. The choice of sealer depends on various factors, including the specific clinical scenario, the clinician’s experience and preference and the desired properties of the material. Regarding the latest advancements, hydraulic calcium silicate-based (HCS) sealers, commonly called “bioceramic sealers”, have gained tremendous popularity in endodontics [6,7,8]. These sealers, especially the fourth and fifth generations, are being intensively investigated due to their relatively recent introduction to the market, their modified chemical composition, their advanced physical and biological properties and the different behavior of these materials in a biological environment [9,10]. All these changes have greatly simplified their clinical applications, even for operators with limited clinical experience [11,12].

For decades, clinicians have successfully used conventional cold or warm (thermoplastic) compaction root canal obturation techniques with a favorable prognosis for endodontically treated teeth [13]. The fundamental principle of these techniques is increasing the gutta-percha volume and minimizing the sealer amount [14]. The development of HCS sealers has significantly changed these principles of root canal obturation. Meanwhile, due to a lack of shrinkage and long-term dimensional stability, these materials can be used in larger volumes for sealer- or filler-based obturations without increasing the amount of gutta-percha in the root canal. Although all obturation techniques are equally effective, the single-cone (SC) obturation technique is easier to apply, especially for inexperienced clinicians [15]. Moreover, the scientific background indicates that the biocompatible, bioactive and antibacterial HCS materials that slightly expand upon setting and remain dimensionally stable in conjunction with a simplified SC obturation can provide much better results than lateral condensation and surpass it as the most efficient endodontic sealing method [16,17].

Endodontic treatment and retreatment, while highly successful, can occasionally present possible complications that require specialized and very complex management [18]. Likewise, specific clinical scenarios may necessitate endodontic surgery to achieve desirable treatment outcomes [19]. Techniques for managing endodontic complications and endodontic surgery have changed and significantly require advanced endodontic materials [11]. Nowadays, HCS materials are considered superior for the management of endodontic complications due to their biological properties, such as biocompatibility, bioactivity and osteogenic potential [10,20]. They are also hydrophilic and hygroscopic, and as they require moisture to set, they are compatible with wet environments like live pulp or periapical tissues [21]. Many materials are currently available for managing endodontic complications, including flowable materials launched as premixed and ready-to-use pastes or powder/liquid formulations [11]. The fourth and fifth types of HCS materials are the most popular in modern endodontics. Both types are tri-calcium silicate-based; however, type four materials are mixed with water, while type five are pre-mixed and ready-to-use [10]. Some materials are only suggested to be used as dentin or root repair materials in conjunction with different application techniques. In contrast, other materials are proposed as sealers or biological fillers and can be used for root canal obturation and root repair. The main advantages of flowable HCS materials are their ease of manipulation and clinical applicability [9].

However, despite all these advantages, they present possible drawbacks. The setting of these materials is based on a hydration reaction, for which an appropriate level of moisture is required [21]. The fourth generation of HCS sealers are water-based materials, and they receive moisture directly from the liquid they are mixed with. Meanwhile, the fifth generation, or premixed formulations, requires environmental moisture [21]. To ensure the complete hydration-based setting of these materials, the root canal should not be overdried [9,21]. Ensuring that the canal is sufficiently dry can be challenging for clinicians, as it is tricky to determine how dry the root canal should be in every clinical case to ensure the complete setting of the premixed HCS. Therefore, further investigations are needed to determine the optimal amount of moisture needed for the materials to set and translate these findings into clinical protocols. Another disadvantage of HCS materials is that water-based formulations are sensitive to heat, and their application when using thermoplastic obturation techniques can significantly change their properties [5]. Therefore, the fourth generation of HCS materials should be used only with cold obturation techniques, while these restrictions are not relevant for premixed formulations [12].

The proper selection of root canal irrigants and efficient irrigation techniques are essential when cleaning and disinfecting the root canal system [22]. Biofilms within the anatomic complexities of the root canal system contain highly resistant bacteria, which are difficult to eradicate with currently available irrigants [22]. Sodium hypochlorite has remained the most widely used irrigant for decades. However, chelators should supplement it to enhance the effectiveness of the irrigation [23], and mixtures of different active substances and nanoparticles have additionally been trialed for this purpose [23]. The delivery of the irrigants using a syringe and needle and their sonic or ultrasonic activation are currently the most popular irrigation methods. At the same time, different agitation and activation methods using negative and positive pressure, or lasers, are widely used and have been investigated to enhance the removal of debris and biofilm from root canal irregularities [24]. Unfortunately, despite these innovations, there is no solid scientific evidence that any adjunct irrigation method can remarkably improve the long-term outcome of root canal treatment [4]. Therefore, redefining this field’s research priorities and strategies, focusing on multidisciplinary scientific approaches and clinically relevant comparisons, is necessary [22].

Some researchers argue that a temporary antibacterial root canal dressing material should be used to enhance root canal disinfection during the endodontic treatment of teeth with pulp necrosis and apical periodontitis, as well as for the management of endodontic complications or regenerative endodontic procedures (REPs) [25]. For decades, calcium hydroxide was the material used for these purposes. Recently, ready-to-use bioceramic-based pastes have been introduced, such as BIO-C^®^ TEMP (Angelus, Londrina, Brazil) and EndoSequence BC Temp (Brasseler USA, Savannah, GA, USA), for intracanal dressing [26]. These materials have antimicrobial properties and are recommended as a substitute for conventional calcium hydroxide dressings, as they are easily washed out of the root canals, eliminating the need for additional irrigation with citric acid, unlike conventional calcium hydroxide paste, which is difficult to remove from the root canal system [27].

Endodontic surgery is indicated when non-surgical endodontic retreatment fails or is impossible. Due to the rapid developments in implant dentistry, endodontic surgery has become a less popular treatment option than tooth replacement with an implant. However, well-designed clinical trials have shown that endodontic retreatment and surgical endodontic procedures are equally effective, if not superior, treatment options [28]. Recently, the fourth and fifth generations of HCS root repair materials have also become increasingly popular for use in endodontic surgery [9,11]. The current scientific findings indicate that these materials have superior properties and handling characteristics and provide healing rates after endodontic surgery similar to those of previously used MTA cement [29,30]. These commercially available plasticized putty-type repair materials are identical in their chemical composition, physical and biological properties and are clinically equally effective [31].

Regenerative endodontics represents a transformative shift in the field of endodontics, focusing on the regeneration of dental pulp tissues and the restoration of tooth vitality [32]. These new biology-based and less invasive concepts have entered the clinical routine, expanding the principles of classical endodontics. REPs, as a biologically based approach, utilize tissue engineering to replace damaged structures, including dentin and cells of the pulp–dentin complex [33]. These procedures aim to heal apical lesions, resolve clinical signs and symptoms, continue root development and strengthen dentin tissue to prevent potential root fractures [25], with the aim of ultimately regenerating a functional pulp–dentin complex and restoring regular nociception. Although stem cells, growth factors, scaffolds and proper disinfection are essential to the overall success of REPs, the biocompatible materials used to cover blood clots play a critical role in the outcome of the treatment [34]. Nowadays, fourth- and fifth-generation HCS materials are recommended for REPs [35].

Pediatric endodontics presents different challenges than adult endodontics, given pediatric patients’ unique anatomical, physiological and behavioral characteristics. The use of appropriate endodontic instruments, materials and techniques in pediatric endodontic cases is crucial to ensuring effective and less time-consuming treatment, patient comfort and a favorable long-term prognosis. Recent material advances in pediatric endodontics address these specific requirements, and a more conservative and minimally invasive approach is usually preferred, particularly vital pulp therapies that preserve dental pulp vitality whenever possible. Nonvital pulp therapy in primary teeth has considerable limitations and is only considered if the pulp is inflamed irreversibly, necrotic and infected, while the roots of the teeth should have minimal or no signs of resorption. The rotary files for pediatric cases were designed for faster root canal shaping, while resorbable injectable filling pastes were proposed for obturation [36]. However, in primary teeth, priority should always be given, whenever possible, to vital pulp therapy based on pulpotomy procedures, including when using hydraulic calcium silicate-based materials [37].

Therefore, this Special Issue, “Advanced Materials for Clinical Endodontic Applications”, aims to deepen researchers’ understanding of the challenges surrounding advanced endodontic materials. However, the current scientific data indicate that there is a critical need to focus on interdisciplinary research that bridges the gap between material science and clinical endodontics. We should translate recent research findings into clinical solutions and protocols to improve endodontic treatment standards and outcomes.
